# Amino acid metabolism, lipid metabolism, and oxidative stress are associated with post-stroke depression: a metabonomics study

**DOI:** 10.1186/s12883-020-01780-7

**Published:** 2020-06-20

**Authors:** Man Wang, Xianwei Gui, Lanxiang Wu, Sheng Tian, Hansen Wang, Liang Xie, Wei Wu

**Affiliations:** Department of Neurology, the Second Affiliated Hospital of Nanchang University, Nanchang, China

**Keywords:** Post-stroke depression, Metabonomics, Liquid chromatography-mass spectrometry, Amino acid metabolism, Lipid metabolism, Oxidative stress

## Abstract

**Background:**

Post-stroke depression (PSD) is a mood disorder characterized by depression and anhedonia caused by stroke. Metabolomics identified metabolites associated with PSD, but previous studies are based on gas chromatography (GC)/mass spectrometry (MS). This study aimed to perform a liquid chromatography (LC)-MS-based metabolomics study of the plasma metabolite profiles between patients with PSD and controls.

**Methods:**

This was a prospective study of patients with stroke enrolled between July and December 2017 at the Second Affiliated Hospital of Nanchang University. Patients were grouped as Hamilton Depression Rating Scale > 7 (PSD) or < 7 (controls). Metabonomics profiling of plasma sampled was conducted by LC-MS. By combining multivariable and univariable statistical analyses, significant differential metabolites between the two groups were screened. The threshold for significant differences was VIP ≥1 and *P* < 0.05. Log_2_FC is the logarithm of the mean ratio between the two groups.

**Results:**

There were no significant difference with respect to age, NIHSS score, and MMSE between the two groups (all *P* > 0.05). There were six differential metabolites between the PSD and stroke groups, of which three metabolites were increased and three were decreased. Compared with the control group, p-chlorophenylalanine (Log_2_FC = 1.37, *P* = 0.03), phenylacetyl glutamine (Log_2_FC = 0.21, *P* = 0.048), and DHA (Log_2_FC = 0.77, *P* = 0.01) levels were higher in the PSD group, while betaine (trimethylglycine) (Log_2_FC = − 0.79, *P* = 0.04), palmitic acid (Log_2_FC = − 0.51, *P* = 0.001), and MHPG-SO_4_ (Log_2_FC = − 2.37, *P* = 0.045) were decreased.

**Conclusion:**

Plasma metabolomics showed that amino acid metabolism (phenylacetyl glutamine, p-chlorophenylalanine, trimethylglycine), lipid metabolism (DHA, palmitic acid, trimethylglycine), and oxidative stress (DHA, palmitic acid, trimethylglycine) were associated with PSD. These results could help to reveal the pathophysiological mechanism of PSD and eventually identify treatment targets.

## Background

Post-stroke depression (PSD) is characterized by depression or severe depressive episode resulting from stroke, with mania or mixed characteristics [1]. The overall incidence of PSD is 31% at any time within 5 years after stroke. PSD can adversely affect neural functional recovery and significantly increase the disability rate and mortality [2]. Therefore, early identification and treatment of PSD are essential to improve prognosis.

At present, the mechanism of PSD remains unclear. According to the monoamine neurotransmitter imbalance hypothesis, brain damage caused by stroke interferes with ascending projection from the brainstem to the frontal cortex through the thalamus and basal ganglia, which results in reduced bioavailability of serotonin, dopamine, and norepinephrine [3]. Some pro-inflammatory cytokines may also affect the synthesis and metabolism of amine neurotransmitters: interferon (INF)-α can affect the synthesis and reuptake of serotonin [4], and interleukin (IL)-1β and tumor necrosis factor (TNF)-α can activate serotonin transporters to increase serotonin reuptake [5].

Beside neurotransmitters, a number of compounds are associated with PSD. Glutamate is closely related to emotional and mental activities. Compared with stroke patients without PSD, stroke patients with PSD have higher glutamate levels in the frontal lobe [6]. Damage to synaptic plasticity in specific regions of the central nervous system, especially the hippocampus, may be a key factor in the pathophysiology of depression. The brain-derived neurotrophic factor (BDNF) plays an important role in the regulation of synaptic plasticity repair [7]. Decreased serum levels of BDNF are associated with PSD [8]. Elevated serum levels of homocysteine (Hcy) are significantly associated with depression [9], whereas Hcy may affect BDNF expression in non-inflammatory states and endothelial dysfunction [10]. The N-acetylaspartate/creatinine ratio in the hippocampus of PSD patients is lower than in controls, suggesting abnormal metabolism of hippocampal neurons in PSD patients [11].

Metabolomics is defined as the study of metabolic networks by analyzing the dynamic changes in the quality and quantity of metabolites of biosystems stimulated by pathology and physiology [12]. Yang et al. have analyzed the serum metabolomics of schizophrenia patients and identified metabolites associated with schizophrenia [13]. Xiao et al. identified five potential biomarkers of PSD: lactic acid, α-hydroxybutyric acid, phenylalanine, formic acid, and arabitol [14]. Zhang et al. found that azelaic acid, glyceric acid, pseudouridine, 5-hydroxycaproic acid, tyrosine, and phenylalanine can be used as biomarkers for the diagnosis of PSD [15]. Ding et al. identified proline, pyroglutamic acid, palmitic acid, oleic acid, linoleic acid, oxalic acid and rhamnose as being associated with PSD [16]. Of note, different methodologies will yield different results simply because they do not look at the same metabolites and those previous studies above are all based on gas chromatography/mass spectrometry (GS/MS).

Liquid chromatography-mass spectrometry (LC-MS) has high sensitivity and requires a simpler sample pretreatment compared with GC/MS [17]. Therefore, the aim of the present study was to perform a LC-MS-based metabolomics study of the plasma metabolite profiles between patients with PSD and controls, in order to identify plasma differential metabolites related to PSD. The results could help identify novel biomarkers of PSD that could ultimately improve prognosis and potentially be treatment targets.

## Methods

### Study design and participants

This was a prospective study of patients with stroke enrolled between July and December 2017 at the inpatient and outpatient clinics of the Department of Neurology of the Second Affiliated Hospital of Nanchang University. This study was approved by the ethics committee of Nanchang University. All patients provided written informed consent to participate in this study. The study was carried out according to the tenets of the Declaration of Helsinki.

The inclusion criteria for the PSD group were: 1) adult patients with confirmed acute stroke (including ischemic and hemorrhagic stroke) within 24 h after onset by computed tomography (CT) or magnetic resonance imaging (MRI); 2) patients meeting 2014 guidelines for the diagnosis of acute ischemic stroke in China; 3) patients meeting the diagnostic criteria of PSD [1]; 4) Hamilton Depression Rating Scale (HDRS; validated Chinese version [18]) > 7; 5) first-ever onset of stroke; 6) no history of antidepressant drugs; and 7) in the acute stage after stroke (< 1 month).

The exclusion criteria were: 1) disturbance of consciousness and/or serious conditions interfering with the examinations; 2) severe dementia, severe aphasia, dysarthria, or deafness; 3) personal or family history of mental disorders; 4) receiving psychiatric treatment; 5) central nervous system infection, head injury, epilepsy, multiple sclerosis, toxic metabolic diseases, Parkinson’s disease, intracranial tumor, or hypothyroidism; 6) discharged within 7 days after onset; 7) death within 2 weeks of onset; or 8) severe metabolic diseases (including severe heart, liver, or renal dysfunction, shock, cancer, autoimmune diseases, etc.).

For the control group, the inclusion and exclusion criteria were the same as for the PSD group, except the HDRS being < 7.

### Plasma sample collection

Venous blood (5 ml) was drawn from an antecubital vein in EDTA anticoagulated vacuum blood collection tubes (BD Medical, Franklin Lake, NJ, USA) after a 12-h fast. The samples were centrifuged at 1500×g for 10 min at 4 °C. The supernatant plasma was pipetted into sterile Eppendorf tubes, 0.5 ml/tube. The samples were snap-frozen in liquid nitrogen for 30 s and stored at − 80 °C.

### Sample preparation

About 50 mg of samples and 20 μL of internal standard solution were added to 1000 μL of extracting solution (methanol:acetonitrile:water = 2:2:1) and vortexed for 30 s. Steel beads were added, a 45-Hz grinder (Shanghai Jingxin Science and Technology Co., Ltd., Shanghai, China) was used for 4 min, and ultrasonic extraction (Shenzhen Lei De Bang Electronics Co., Ltd., Shenzhen, China) was performed in ice for 5 min. The extraction were repeated for three times and the samples were left standing 1 h at − 20 °C. After centrifugation at 13,000 rpm for 15 min at 4 °C, 700 μL of the supernatant was taken in an EP tube, dried in a vacuum concentrator, and re-dissolved with 500 μL of acetonitrile:water: 1:1. The sample was vortexed for 30 s and treated in the ultrasonic bath for 10 min. After centrifugation at 13,000 rpm for 15 min at 4 °C, 60 μL of the supernatant was taken out in a 2-mL injection bottle for LC-MS analysis. The quality control (QC) samples were prepared by mixing the experimental sample extracts in equal amounts to analyze the repeatability of the sample under the same treatment method. During analysis, a QC sample was inserted every 6–10 test samples to monitor repeatability.

### LC-MS analysis

The instrument platform for LC-MS analysis included a 1290 Ultra High Performance Liquid Chromatography (Agilent Technologies, Santa Clara, CA, USA) and a Triple TOF 6600 High Resolution Mass Spectrometer (AB SCIEX LLC, Framingham, MA, USA). The column was a UPLC HSS T3 chromatographic column (1.7 μm, 2.1 × 100 mm, Waters, Milford, MA, USA). Mobile phase A was water (containing 25 mM ammonium acetate and 25 mM ammonia water) and mobile phase B was acetonitrile. The gradient elution procedure is shown in Table 1. The injection volume was 1 μL.

**Table 1:** Parameters of the mobile phase for LC-MS

Time (min)	Row (μL/min)	A%	B%
0	500	5	95
0.5	500	5	95
7	500	35	65
8	500	60	40
9	500	60	40
9.1	500	5	95
12	500	5	95

For MS, data acquisition was under the control of the control software (Analyst TF 1.7, AB SCIEX LLC, Framingham, MA, USA). In each cycle of data acquisition, molecular ions with the highest intensity and greater than 100 were screened for the acquisition of corresponding secondary mass spectral data. Bombardment energy: 30 eV, 15 secondary spectrograms per 50 ms. The ESI ion source parameters were set to: atomization pressure (GS1): 60 Psi; auxiliary pressure: 60 Psi; air curtain pressure: 35 Psi, temperature: 650 °C, spray voltage: 5000 V (positive ion mode) or − 4000 V (negative ion mode) [19, 20].

### Data analysis

The plasma metabolite profiles in the PSD and control groups were analyzed. A multidimensional statistical model was established to screen for metabolites with significant differences between the two groups, and to identify biologically significant metabolites related to PSD. The original data after MS were first converted to the mzML format using the ProteoWizard software. XCMS 1.5.0 was used for data processing, such as retention time correction, peak identification, peak extraction, peak integration, and peak alignment. Minfrac was set as 0.5 and cutoff as 0.6. The xcms4dda and xcms4lipid software and a self-built library developed based on XcmMS were used for data filtering. The filtering criterion was that sample number of the metabolite in at least one group of samples was detected to be greater than 50% (minfrac = 0.5).

### Model identification

Principal component analysis (PCA), partial least squares-discriminant analysis (PLS-DA), and orthogonal partial least-squares discriminant analysis (OPLS-DA) were used. PCA analysis represents an unsupervised multidimensional statistical analysis method that can generally reflect the overall metabolic differences among groups and the degree of variation within groups. PLS-DA and OPLS-DA are statistical analysis methods with supervised pattern recognition that can maximize the distinction between groups, thus contributing to the search for differential metabolites (potential biomarkers). Given multivariable statistical analysis would have over-fitting, the multivariable statistical analysis model also needed to undergo model verification, permutation test was used to evaluate the accuracy of the (O)PLS models. By combining the multivariable statistical analysis (OPLS-DA variable important in projection (VIP) values) and univariable statistical analyses (Student’s t-test), significant differential metabolites between the two groups were screened. The threshold for significant difference was: VIP ≥1 and t-test *P* < 0.05. The metabolite-related pathways were searched for using KEGG (http://www.kegg.jp).

### Statistical analysis

Continuous data are presented as means ± standard deviation and were analyzed using the Student t test. Categorical data are presented as frequencies and were analyzed using the chi-square test. All statistical analyzes were performed using SPSS 16.0 (IBM, Armonk, NY, USA). Two-sided *P* values < 0.05 were considered statistically significant.

## Results

### Participants

Ten stroke patients without PSD and 10 stroke patients with PSD were recruited. There were no significant differences with respect to age, NIHSS score, and MMSE between the two groups (Table 2).

**Table 2:** Characteristics of the participants

	Control	PSD	P
Age (years) Gender (male/female)	57.9 ± 9.7 9/1	61.1 ± 14.4 7/3	0.35 0.58
HDRS	2.0 ± 2.1	15.1 ± 5.4	< 0.001
NIHSS	3.6 ± 4.4	2.8 ± 3.7	0.39
MMSE	26.3 ± 4.4	23.4 ± 6.5	0.48

*HDRS* Hamilton Depression Rating Scale; *NIHSS* National Institutes of Health Stroke Scale; *MMSE* Mini-Mental State Examination

### Chromatograms

Visual examination on the total ion current (TIC) of all samples was conducted. TIC is a chromatogram of the sum of all ionic strengths and the time in the selected mass range. TIC is a reflection of category and abundance of the whole metabolites of samples. The analysis of all samples showed good overlapping and stability of TIC peak retention time and peak area, indicating the reliability of the chromatograms.

### Principal component analysis (PCA)

**Fig. 1:**
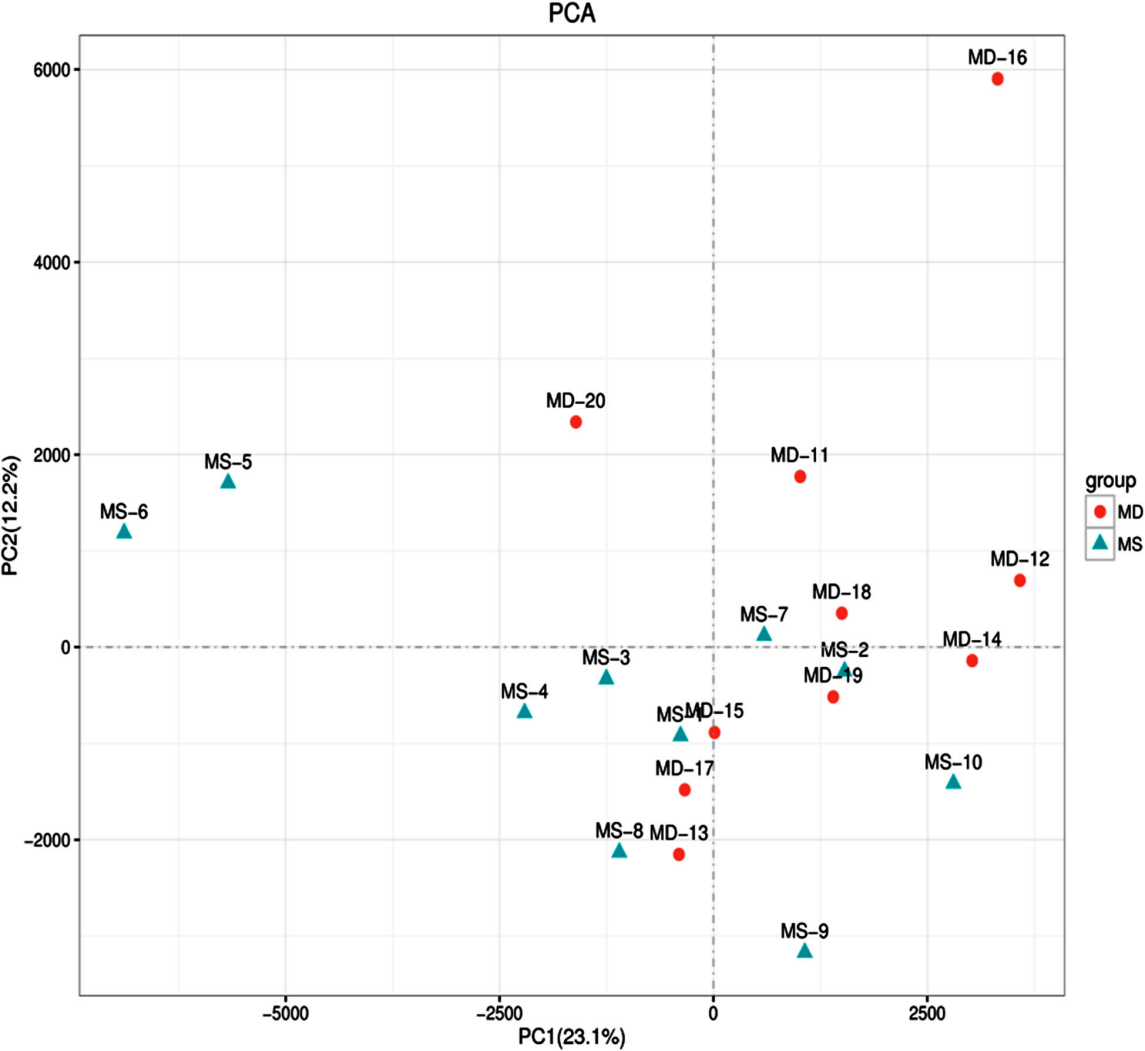
Principal component analysis scores plot of plasma from the post-stroke depression and control group. Each dot represents an individual sample

The PCA model could explain the metabolic differences between the PSD and control groups (Fig. 1).

### Partial least squares-discriminant analysis (PLS-DA)

**Fig. 2:**
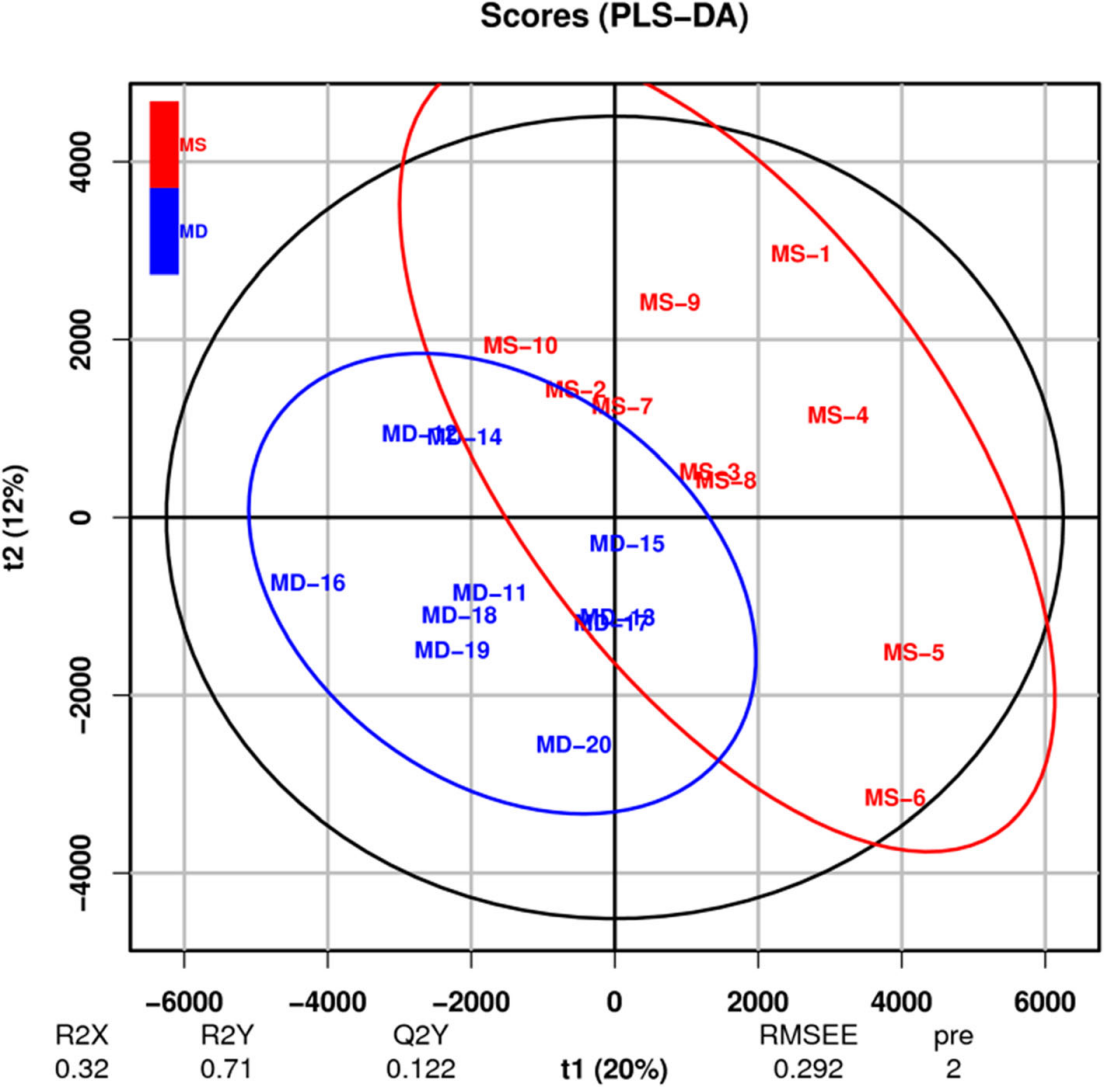
PLS-DA scores plot of plasma from the post-stroke depression and control groups. Each dot represents an individual sample

PLS-DA is a multivariable statistical analysis method with supervised pattern recognition. The multi-dimensional data undergo grouping according to the differential factors that need to be found before compression (the Y value can be pre-set for target classification and discrimination), so variables that are the most relevant to grouping factors can be found, while reducing the impact of the other factors. Similar to the PCA analysis, PLS-DA can maximize the distinction between groups, which is helpful to search for differential metabolites. The VIP for a model variable can measure the influence intensity and interpretability of accumulated differences for each metabolite on the classification and discrimination of samples in each group. VIP ≥1 is a common screening standard for differential metabolites. Figure 2 shows that samples in the two groups are on the left and right sides of the PLS-DA score map, respectively, indicating significant metabolic differences of samples between the two groups.

### Orthogonal partial least-squares discriminant analysis (OPLS-DA)

**Fig. 3:**
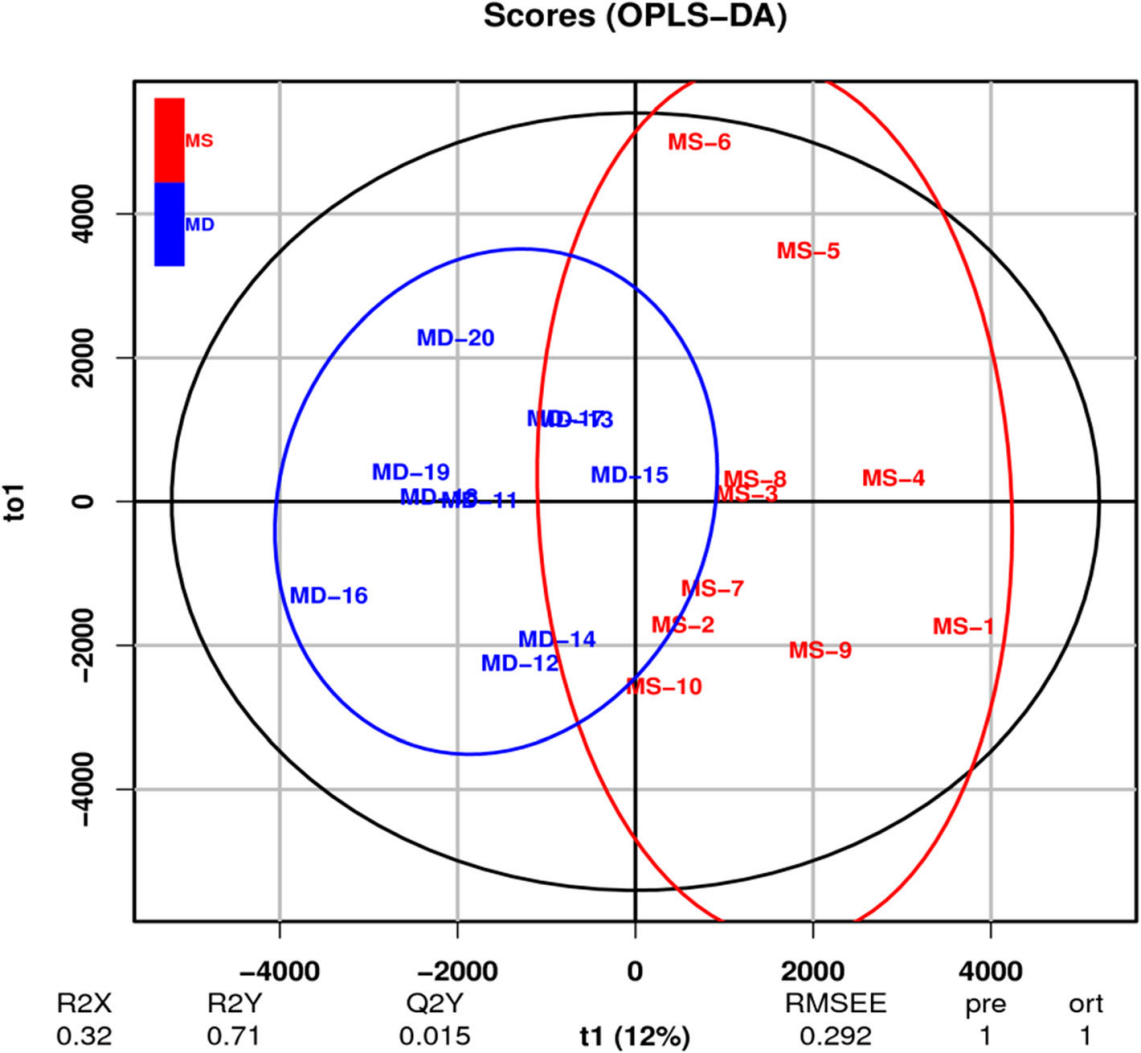
OPLS-DA scores plot of plasma from the post-stroke depression and control groups. Each dot represents an individual sample

OPLS-DA is a derivative algorithm of PLS-DA. Compared with PLS-DA, OPLS-DA integrates orthogonal signal correction (OSC) and PLS-DA, decomposing X matrix information into two types of information that are related and unrelated to Y. By removing unrelated differences, the related information is concentrated in the first predictive component. Subsequent model tests and screening of differential metabolites were analyzed with the use of OPLS-DA results. Figure 3 shows that the samples in the two groups are on the positive and negative sides of the first principal component, indicating that samples in the two groups have significant metabolic differences on the OPLS-DA score map.

### Plasma differential metabolites

When combining multivariable statistical analyses (OPLS-DA VI*P* values) and univariable statistical analyses (t test P values), significant differential metabolites between the two groups were screened. The threshold for significant differences was VIP ≥1 and *P* < 0.05.

There were six differential metabolites between the PSD and stroke groups, of which three metabolites were increased and three were decreased. Compared with the control group, p-chlorophenylalanine, phenylacetyl glutamine, and DHA levels were higher in the PSD group, while betaine (trimethylglycine), palmitic acid, and MHPG-SO_4_ were decreased (Table 3).

**Table 3:** Differential metabolites between the post-stroke depression and control groups

#	Compounds	RT	M/Z	VIP (OPLS-DA)	P (t-test)	log_2__FC
1	α-N-Phenylacetyl-L-glutamine	202.24	265	2.11	0.028	1.37
2	IS	233.88	200	2.63	0.048	0.21
3	DHA	36.67	327.23	1.47	0.011	0.77
4	Betaine	286.68	118.08	1.43	0.043	−0.79
5	Palmitic acid	73.98	274.27	6.35	0.001	−0.51
6	MHPG-SO_4_	32.31	282.28	2.98	0.045	−2.37

Log_2_FC is the logarithm of the mean ratio between the stroke group and post-stroke depression group. Positive numbers represent increase in the post-stroke depression group compared with the control group, and negative numbers mean a decrease

### KEGG analysis

Table 4 presents the pathways involving the differential metabolites identified above between the 10 stroke patients without PSD and 10 stroke patients with PSD. Palmitic acid is involved in the fatty acid elongation, fatty acid degradation, fatty acid metabolism, fatty acid biosynthesis, and biosynthesis of unsaturated fatty acids pathways. Betaine is involved in the glycine, serine, and threonine pathway. α-N-Phenylacetyl-L-glutamine is involved in the phenylalanine metabolism pathway.

**Table 4:** KEGG pathway analysis

#	Pathway	Pathway ID	C_id	Differentially expressed metabolites	Description
1	Fatty acid elongation	ko00062	C00249	1021	Hexadecanoic acid; Hexadecanote; Hexadecylic acid; Palmitic acid; Palmitate; Cetylic acid
2	Fatty acid degradation	ko00071	C00249	1021	Hexadecanoic acid; Hexadecanote; Hexadecylic acid; Palmitic acid; Palmitate; Cetylic acid
3	Fatty acid metabolism	ko01212	C00249	1021	Hexadecanoic acid; Hexadecanote; Hexadecylic acid; Palmitic acid; Palmitate; Cetylic acid
4	Glycine, serine and threonine metabolism	ko00260	C00719	90	Betaine; Trimethylaminoacetate; Glycine betaine; N,N,N-Trimethylglycine; Trimethylammonioacetate
5	Phenylalanine metabolism	ko00360	C04148	945	Phenylacetylglutamine; alpha-N-Phenylacetyl-L-glutamine; N2-(2-Phenylacetyl)-L-glutamine
6	Fatty acid biosynthesis	ko00061	C00249	1021	Hexadecanoic acid; Hexadecanote; Hexadecylic acid; Palmitic acid; Palmitate; Cetylic acid
7	Biosynthesis of unsaturated fatty acids	ko01040	C00249	1021	Hexadecanoic acid; Hexadecanote; Hexadecylic acid; Palmitic acid; Palmitate; Cetylic acid

## Discussion

In the present study, LC-MS-based metabolomics was used to analyze plasma metabolite profiles in PSD patients, and the metabolites related to PSD were screened. The KEGG was conducted for further analysis of metabolic pathways and the results suggest that plasma differential metabolites were predominantly related to amino acid metabolism (phenylalanine-tyrosine, tryptophan, methionine, and Hcy metabolisms), lipid metabolism, and oxidative stress.

Different methodologies will yield different results simply because they do not look at the same metabolites and those previous studies above are all based on GS/MS. Indeed, Xiao et al. identified five potential biomarkers of PSD: lactic acid, α-hydroxybutyric acid, phenylalanine, formic acid, and arabitol [14]. Zhang et al. found that azelaic acid, glyceric acid, pseudouridine, 5-hydroxycaproic acid, tyrosine, and phenylalanine can be used as biomarkers for the diagnosis of PSD [15]. Ding et al. identified proline, pyroglutamic acid, palmitic acid, oleic acid, linoleic acid, oxalic acid and rhamnose as being associated with PSD [16]. The differences between those three previous studies and the present study might be due to the sample preparation process and detection methods.

The differential metabolites associated with amino acid metabolism included phenylacetyl glutamine, p-chlorophenylalanine, and trimethylglycine (betaine). Compared with the stroke group, levels of phenylacetyl glutamine and p-chlorophenylalanine were increased, while the levels of trimethylglycine were decreased in the PSD group.

Phenylacetyl glutamine is an important metabolite of phenylalanine. There are two main metabolic pathways for phenylalanine in human body: 1) phenylalanine transaminase catalyzes phenylalanine to phenylpyruvic acid, ultimately generating phenylacetyl glutamine; and 2) phenylalanine hydroxylase catalyzes phenylalanine to tyrosine. Tyrosine can be used to synthesize neurotransmitters such as dopamine and norepinephrine, which are directly related to the onset of depression. The up-regulation of phenylacetyl glutamine in the PSD group may be associated with imbalance of the two metabolic pathways of phenylalanine in the PSD group, with more phenylalanine being catalyzed into phenylacetyl glutamine rather than tyrosine, leading to depressive symptoms. Based on GC-MS urine metabolomics, Zhang et al. found that levels of tyrosine and phenylalanine were decreased in the PSD group [15], supporting the present study and consistent with metabolic disorder of phenylalanine in the PSD.

Tryptophan is converted to serotonin via oxidative decarboxylation by tryptophan hydroxylase [21]. Tryptophan hydroxylase can be selectively inhibited by p-chlorophenylalanine, resulting in a reduction in synthesis of serotonin [22]. Therefore, the up-regulation of p-chlorophenylalanine levels in PSD in the present study may indirectly indicate a decrease in serotonin level in PSD.

Betaine is a highly active methyl donor and an alkaloid of quaternary ammonium salt, which is involved in the methionine-Hcy circulation. Betaine transfers methyl to Hcy through the betaine-Hcy methyltransferase (BHMT) in the liver, transformed into methionine and dimethylglycine, replacing methionine to provide methyl groups for other metabolic processes [23]. Methionine can react with ATP to form S-adenosylmethionine (SAM), which is the only methyl donor in the central nervous system, and it can promote the metabolism of neurotransmitters such as dopamine, serotonin, and norepinephrine [24]. Therefore, when betaine levels are decreased, due to the decrease of methyl donor, an increase in Hcy level, and a reduction in the production of methionine, the generation of dopamine, serotonin, and norepinephrine is reduced, resulting in depression. In addition, Hcy interferes with brain functions due to direct toxic effects on cerebral vascular endothelial cells, and it has toxic effects on depression-related nerve cells through various pathways, leading to depressive symptoms [24–26].

Among the differential metabolites screened, the KEGG analysis identified that the fatty acid elongation, fatty acid degradation, fatty acid metabolism, fatty acid biosynthesis, biosynthesis of unsaturated fatty acids pathways, the glycine, serine, and threonine pathway, and the phenylalanine metabolism pathways were probably involved in some manner in PSD. Nevertheless, those pathways are very complex and involve a number of metabolites, often intracellular, that were not detected in the present study. Additional studies are necessary to determine how those pathways are involved in PSD.

DHA is a long-chain ω-3 polyunsaturated fatty acid and is associated with depression in many aspects. First, at the level of cell signal transduction, DHA is the predominant component of long-chain unsaturated fatty acids in cell membrane phospholipids, maintaining stability and fluidity of cell membrane, which plays an important role in cell signal transduction [27]. DHA has an effect on central neurotransmitters, especially the neural transmission or functions of serotonin and dopamine. Studies have shown that dopamine release in the brain regions of rats without long-chain ω-3 polyunsaturated fatty acids is significantly lower than in controls [28]. Epidemiological data have demonstrated that small intake of fish products in depression patients is directly related to the depression, and that intake of long-chain ω-3 polyunsaturated fatty acids is associated with a lower risk of suicide [29].

Second, DHA has an important effect on PPAR signaling pathways in inflammation. DHA can increase the expression levels and protein activity of PPAR-γ, and PPAR-γ can inhibit NF-κB pathway after its activation, reducing the inflammatory response and promoting cell metabolism and mitochondrial function [30, 31]. Studies have shown that mitochondrial morphological and functional abnormalities caused by oxidative stress may be related to depression, and mitochondrial dysfunction resulting from oxidative stress can change intracellular metabolism; lipid peroxidation is a notable metabolic characteristic in mitochondrial metabolic disorders, which is related to the psychopathology of MDD [32–34]. Oxidative stress occurs after stroke, and brain cells will consume a large amount of oxygen, producing massive amounts of free radicals. Given that brain tissue contains less antioxidants, high-density membrane unsaturated fatty acids, and higher oxidative metabolism rate, it is prone to be damaged by reactive oxygen species [35]. DHA can increase the levels of antioxidant enzymes, and by modifying the activity of superoxide dismutase (SOD), it can eliminate free radical produced after stroke and has a protective effect on the brain [36]. In the present study, the levels of DHA were higher in the PSD group compared with the control group. It is speculated that DHA in the PSD group is more effective in anti-oxidative stress injury after stroke than in promoting the release of neurotransmitters such as serotonin and dopamine. Ding et al. found that linoleic acid (a long-chain ω-6 polyunsaturated fatty acid) and oleic acid (a monounsaturated ω-9 fatty acid) were reduced in PSD based on GC-MS analysis [16]. Nevertheless, the association between metabolism disorders of unsaturated fatty acid and post-stroke depression needs further exploration.

Palmitic acid is the free saturated fatty acid with the highest level in plasma. Excessive consumption of fatty acids is often encountered in stroke patients, which is a reaction of restoring bio-energy balance state initiated by the protective regulatory system of the central nervous system when a decline in energy occurs due to stroke [37]. On the other hand, studies have shown that palmitic acid can activate NF-κB, promote massive release of inflammatory cytokines, and induce inflammatory responses of macrophages [38]. NF-κB is a key factor in connecting oxidative stress and inflammation [39]. In the present study, it was found that the plasma levels of palmitic acid were decreased in the PSD group, which was consistent with previous studies based on GC-MS methods [39]. Further research is needed to explore whether lower levels of palmitic acid is associated with related pathways of inflammation and oxidative stress, causing depressive symptoms, or lower levels are related to massive consumption and energy supply of fatty acids in PSD.

In amino acid metabolism (described above), betaine, as a methyl donor, is involved in the metabolism of methionine and Hcy, which may play a role in PSD. In addition, betaine, as a methyl donor, can also enhance carnitine synthesis and promote β-oxidation of long-chain fatty acids [40]. Some studies also showed that betaine can increase the activity of GSH-PX and SOD, eliminate oxygen free radicals, and play a protective role against oxidative stress damage of hippocampal neurons [13]. Multiple aspects could explain the decrease in betaine levels in the post-stroke depression group in this study and additional research is necessary to explain this.

The last differential metabolite was MHPG-SO_4_, which showed a decrease in plasma levels in the PSD group compared with the control group. The main metabolite of norepinephrine in the brain is MHPG and it is combined with a sulfate group for urine excretion. In this study, the decrease in MHPG-SO_4_ levels in the PSD group could possibly be explained by a decrease in the concentration of adrenaline at synaptic cleft and a reduction in metabolism in PSD patients, further supporting the monoamine neurotransmitter hypothesis in PSD patients. Xiao et al. showed that compared with controls, the levels of plasma MPHG were significantly reduced in patients with depression before treatment, and MHPG levels showed an increase after effective antidepressant treatment [41].

The present study has limitations. Only 10 patients were included in each group, and the sample size was small. Nevertheless, due to stricter HDRS score for enrolled PSD patients, the smaller sample size was appropriate for metabonomics analyses. Therefore, larger sample size and multicenter studies are still needed to determine the biomarkers of PSD.

## Conclusions

In the present study, metabolomics based on LC-MS was used to analyze the plasma metabolites in PSD patients. Six differential metabolites were screened, including phenylacetyl glutamine, p-chlorophenylalanine, betaine, DHA, palmitic acid and MHPG-SO_4_, suggesting that there are amino acid and lipid metabolism disorders in PSD patients, as well as increased oxidative stress in PSD.

## Data Availability

The datasets used and/or analyzed during the current study are available from the corresponding author on reasonable request.

## Abbreviations

BDNF: Brain-derived neurotrophic factor
BHMT: Betaine-Hcy methyltransferase
CT: Computed tomography
GS/MS: Gas chromatography/mass spectrometry
Hcy: Homocysteine
HDRS: Hamilton depression rating scale
IL: Interleukin
INF: Interferon
LC-MS: Liquid chromatography-mass spectrometry
MRI: Magnetic resonance imaging
OPLS-DA: Orthogonal partial least-squares discriminant analysis
OSC: Orthogonal signal correction
PCA: Principal component analysis
PLS-DA: Partial least squares-discriminant analysis
PSD: Post-stroke depression
QC: Quality control
SAM: S-adenosylmethionine
SOD: Superoxide dismutase
TIC: Total ion curren
TNF: Tumor necrosis factor
VIP: Variable important in projection
